# Natural evolution of desmoplastic fibroblastoma on magnetic resonance imaging: a case report

**DOI:** 10.1186/1752-1947-5-139

**Published:** 2011-04-07

**Authors:** Yusaku Kamata, Ukei Anazawa, Hideo Morioka, Takeshi Morii, Keiko Miura, Makio Mukai, Hiroo Yabe, Yoshiaki Toyama

**Affiliations:** 1 Department of Orthopaedic Surgery, Keio University, 35 Shinanomachi, Shinjuku-ku, Tokyo 160-8582, Japan; 2 Department of Orthopaedic Surgery, Kyorin University, 6-20-2 Shinkawa, Mitaka, Tokyo 181-8611, Japan; 3 Department of Diagnostic Pathology, Keio University Hospital, 35 Shinanomachi, Shinjuku-ku, Tokyo 160-8582, Japan

## Abstract

**Introduction:**

Desmoplastic fibroblastoma (collagenous fibroma) is a recently described tumor thought to arise predominantly from subcutaneous tissue or skeletal muscle. The natural evolution of this tumor on magnetic resonance imaging has never been described, to the best of our knowledge. We herein report a case of desmoplastic fibroblastoma arising in the thigh and show the longitudinal magnetic resonance imaging findings.

**Case presentation:**

A 60-year-old Japanese man presented with swelling of the medial side of his right thigh, and he complained of nighttime pain and slight tenderness. Magnetic resonance imaging demonstrated a 4 × 4 cm mass in the right thigh. Open biopsy was performed. The mass was diagnosed histologically as a benign fibrous tumor, and we maintained follow-up without surgical therapy. After one year, magnetic resonance imaging showed an increase in tumor size to 4 × 5 cm, but the histologic findings were the same as those obtained one year earlier. Resection was performed with narrow surgical margins. Pathologic diagnosis was desmoplastic fibroblastoma. Two years after surgery, the patient is free from pain and shows no signs or symptoms of recurrence.

**Conclusion:**

The natural evolution of desmoplastic fibroblastoma is characterized by no changes in patterns on magnetic resonance imaging despite increasing size. This finding is clinically helpful for distinguishing desmoplastic fibroblastoma with increasing pain from the desmoid tumor.

## Introduction

In 1995, Evans [1] first described desmoplastic fibroblastoma, a unique fibrous soft tissue tumor comprising spindle-shaped to stellate fibroblastic cells sparsely distributed in a dense fibrous background. This tumor, alternatively called *collagenous fibroma *[2], was clinically and morphologically distinct, as well as completely benign in previously reported series. Details of magnetic resonance imaging (MRI) findings for this tumor have been described for three cases [3,4], but the natural evolution of this tumor has never been described. Here, we present the natural evolution of this tumor on MRI.

## Case presentation

A 60-year-old Japanese man presented with swelling of the medial side of his right thigh and complained of night pain and slight tenderness. Palpation disclosed a hard, well-circumscribed, mobile tumor. Otherwise healthy, he had no history of trauma to the thigh. The results of routine laboratory studies were normal.

**Figure 1:**
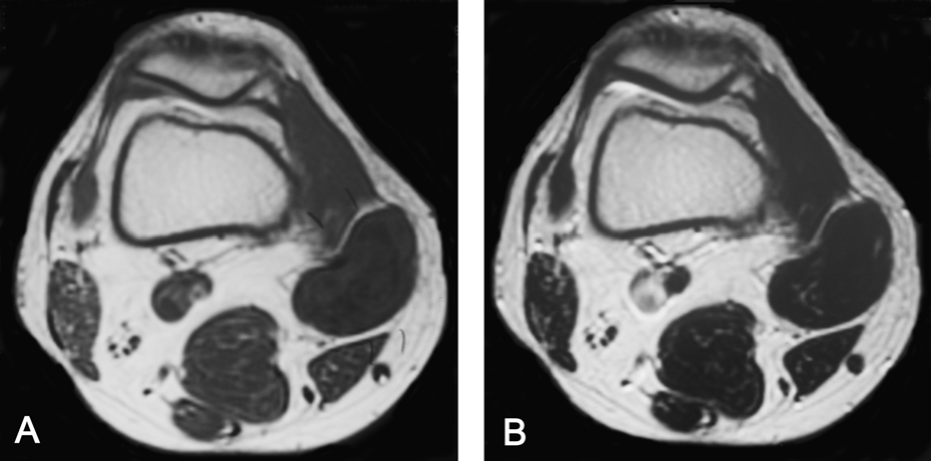
**Magnetic resonance imaging findings, July 2001**. **A) **Axial T1-weighted image (TR/TE: 500/14) showing a 4 × 4 cm, well-circumscribed, inhomogeneous mass as predominantly isointense with muscle containing areas of low signal intensity (SI). **B) **Axial T2-weighted image (TR/TE: 4000/105) showing scattered areas of high SI within low overall SI.

**Figure 2:**
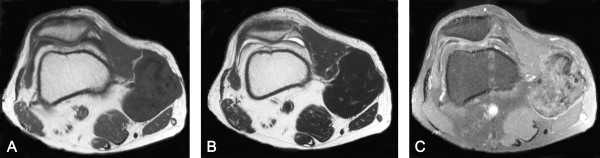
**Magnetic resonance imaging findings, April 2002**. **A) **Axial T1-weighted image (TR/TE: 500/8.6) showing an increasing size and the same pattern as one year previously. **B) **Axial T2-weighted image (TR/TE: 4000/104) showing the same ratio of high signal intensity (SI) area as in previous images. **C) **Axial postcontrast T1-weighted image (TR/TE: 500/8.6) showing nonenhanced areas that correspond to those showing low SI in precontrast T1-weighted images.

MRI demonstrated a 4 × 4 cm mass in the right thigh, occupying a space between the vastus medialis, sartorius and semimembranosus muscles (Figure 1A, B). Open biopsy was performed. Because the mass was diagnosed histologically as a benign fibrous tumor, we maintained follow-up without surgical therapy. After one year, MRI showed an increase in tumor size to 4 × 5 cm. T1-weighted images characterized the mass as well-circumscribed and inhomogeneous, with signals predominantly isointense with muscle, but including several areas of low signal intensity (SI) (Figure 2A). On T2-weighted images, the mass predominantly showed low SI with scattered high-SI areas (Figure 2B). These MRI findings were the same as those obtained one year previously except for tumor size. T1-weighted images after contrast agent administration revealed heterogeneous enhancement of the lesion. Nonenhanced areas corresponded to regions with low SI on noncontrast T1-weighted images (Figure 2C). Based on this growth, with pain and enhancement of the lesion, resection was performed with narrow surgical margins; the tumor adjoined the vastus medialis, sartorius and semimembranosus muscles and was adjacent to Hunter's canal with fibrous adhesion to the adductor magnus tendon.

**Figure 3:**
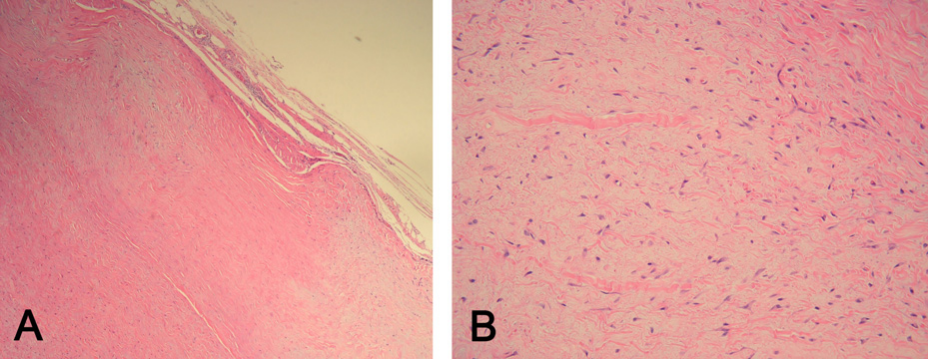
**Pathologic findings (hematoxylin and eosin stain)**. **A) **Low-power photomicrograph of the tumor demonstrating fibrous and myxocollagenous areas. The tumor border is well defined. Fibrous area demonstrates wavy and dense collagen fibers. Cellularity is very low, consisting of spindle-shaped and stellate tumor cells. **B) **High-power photomicrograph of the area showing a myxocollagenous stroma, demonstrating benign-appearing spindle cells presenting higher cellularity than in the fibrous area.

Pathologically, the tumor showed well-circumscribed borders and low vascularity (Figure 3A). Spindle-shaped and stellate fibroblastic cells were sparsely distributed in a dense fibrous background. In some myxocollagenous areas, cellularity was greater than in the fibrous area (Figure 3A, B). No mitotic figures were seen. The pathologic diagnosis was desmoplastic fibroblastoma.

At the most recent follow-up examination two years after surgery, the patient was free from pain and showed no signs or symptoms of recurrence.

## Discussion

Evans [1] used the designation *desmoplastic fibroblastoma *when he described seven patients having masses with distinct morphologic characteristics. Subsequently, Nielsen *et al. *[2] reported seven other cases and proposed that the tumor be renamed *collagenous fibroma*, arguing that *desmoplastic fibroblastoma *misleads the reader into believing that the lesion consists of immature tumor cells inducing a desmoplastic response in host tissues.

These neoplasms typically occur in the fifth or sixth decades of life and occur two and a half times more often in men than women. Clinically, desmoplastic fibroblastoma presents as a firm, mobile, slowly growing mass located in subcutaneous tissue or near the deep aspect of skeletal muscles. Common locations include the arm, shoulder, posterior neck and upper back [1-8]. Few previous reports have described tumors arising in the thigh [1,2,6,8]. In the present patient, the tumor appeared to arise from the fascia of the thigh muscles. Such tumors located in the intermuscular space have not been described. The mass is usually painless; however, our patient complained of pain, as have those in some previous reports [3,5,6]. In our case, location of the tumor adjacent to Hunter's canal may have contributed to pain by compressing the saphenous nerve in the canal.

Previous authors recommended treatment of desmoplastic fibroblastoma by total surgical excision. No tumor recurrence during follow-up has been reported, including the present case.

On MRI, T1-weighted images of the mass depicted a mixture of low SI and isointensity. With T2-weighting, the mass showed scattered high-SI areas within a zone of low SI. Contrast T1-weighted images demonstrated inhomogeneous enhancement after contrast administration, and nonenhanced areas corresponding to regions showing low SI on noncontrast T1-weighted images. The size of the tumor increased over the course of one year, but the MRI findings of the tumor showed no changes.

Several reports have described the MRI appearance of this tumor, but in only three cases have MRI findings been described in detail [3,4], and those findings have varied. T1-weighted findings were described as having diverse SI, low SI compared with muscle, scattered isointense areas [4], and isointense areas with scattered areas of low and high SI [3]. T2-weighted findings also have varied among cases, including high-SI areas with small low-SI areas [4] and low-SI areas with small high-SI areas [3]. Postcontrast T1-weighted images have only been described in two cases. They showed enhanced areas within areas of low SI, and enhanced areas showed strong enhancement in one case, with inhomogeneous enhancement seen in the other case [3,4].

In previous reports, the relationship between MRI findings and histologic findings has been described. On T2-weighted images, low-SI areas correspond to abundant collagen fibers [3,4,9], and high-SI areas showing marked enhancement on contrast T1-images correspond to those histologically consisting of fibroblasts and loose collagen fibers [4]. On T1-weighted images, low-SI areas represent areas with low cellularity and abundant collagen fibers [3].

These reports indicate that MRI findings vary among and within individual tumors because of the variable cellularity.

Evans [1] suggested that the most significant differential diagnostic consideration was desmoid tumor because it may have similar cytologic features and is often locally aggressive.

On the other hand, Marco *et al. *[10] reported that desmoplastic fibroblastoma is a myofibroblastic lesion ultrastructurally demonstrating the presence of fibronexus junctions; markers of myofibroblastic differentiation, typically present on the cytoplasmic membrane of the cells, while desmoid tumor is fibroblastic. This ultrastructural finding is important in the differential diagnosis between desmoplastic fibroma and desmoid tumor.

Details of imaging studies of desmoid tumors have been well documented in previous reports [11-13], and the margins of the tumor are mostly ill defined. However, some MRI findings of desmoid tumor are similar to those of desmoplastic fibroblastoma [11,12]. Several reports have described desmoid tumors as resulting in pain [14], and Agrawal *et al. *[15] reported an increasing desmoid tumor with pain. When desmoplastic fibroblastoma increases with pain, such as in our case, the tumor needs to be distinguished from desmoid tumor.

The natural evolution of desmoid tumor has been documented [13], but that of desmoplastic fibroblastoma has not been previously reported. Vandevenne *et al. *[13] described the MRI findings of the natural evolution of desmoid tumors. Desmoid tumors showing high SI on T2-weighted images tend to increase in size. Subsequently, they show an increase in areas of low SI on T2-weighted images. Finally, they acquire an overall low SI both on T1- and T2-weighted images and decrease in size. In contrast, the MRI findings of desmoplastic fibroblastoma in our case showed an increase in tumor size, with the same pattern: mainly low SI on T1-weighted images. The follow-up period of one year and the two sets of MRIs may not be sufficient to understand the true behavior of slow-growing tumors, such as desmoplastic fibroblastoma. Clinically, however, changes in MRI findings provide important information in distinguishing between aggressive and slow-growing tumors.

## Conclusion

This case study has described the MRI findings of desmoplastic fibroblastoma in relation to its natural evolution. The natural evolution of desmoplastic fibroblastoma is characterized by no change in patterns on MRI despite increasing size. This finding is clinically helpful for distinguishing desmoplastic fibroblastoma with increasing pain from desmoid tumor.

## Competing interests

The authors declare that they have no competing interests.

## Consent

Written informed consent was obtained from the patient for publication of this case report and accompanying images. A copy of the written consent is available for review by the Editor-in-Chief of this journal.

## Authors' contributions

YK drafted the manuscript and reviewed the literature. UA supervised treatment of the patient, carried out the patient's surgery and revised the manuscript. MM performed the histopathological analysis and helped draft the manuscript. HM, TM, KM, HY and TY helped draft the manuscript. All authors have read and approved the final manuscript.
